# Crystal structure of ethyl (*E*)-2-cyano-3-(thio­phen-2-yl)acrylate: two conformers forming a discrete disorder

**DOI:** 10.1107/S2056989017010799

**Published:** 2017-08-04

**Authors:** Brian Castro Agudelo, Juan C. Cárdenas, Mario A. Macías, Cristian Ochoa-Puentes, Cesar A. Sierra

**Affiliations:** a Departamento de Química, Universidad Nacional de Colombia, Bogotá D.C., Colombia; b Departamento de Química, Universidad de los Andes, Carrera 1 No 18A-12, Bogotá D.C., Colombia

**Keywords:** crystal structure, thio­phene-based cyano­acrylate derivatives, mol­ecular disorder

## Abstract

In the title compound, C_10_H_9_NO_2_S, all the non-H atoms, except for the ethyl fragment, lie nearly in the same plane. Despite the mol­ecular planarity, the ethyl fragment presents more than one conformation, giving rise to a discrete disorder, which was modelled with two different crystallographic sites for the eth­oxy O and eth­oxy α-C atoms, with occupancy values of 0.5. In the crystal, the three-dimensional array is mainly directed by C—H⋯(O,N) inter­actions, giving rise to inversion dimers with *R*_2_^2^(10) and *R*_2_^2^(14) motifs and infinite chains running along the [100] direction.

## Abstract 2

The mol­ecular structure of the title compound is characterized by a planarity that allows the formation of (010) sheets. However, the existence of two different conformations of the ethyl fragment introduces the occurrence of discrete disorder due to a mol­ecular overlay.

## Chemical context   

Cyano­acrylate derivatives are organic compounds with a very important industrial inter­est due to their use as monomers in the production of adhesives and polymer materials (Gololobov & Krylova, 1995 ▸). Furthermore, these compounds have been described as promissory inter­mediates for heterocycle synthesis (Gololobov *et al.*, 1995 ▸) and as nitrile-activated precursors in bioreduction reactions (Winkler *et al.*, 2014 ▸). Still, their most outstanding application is related to their very attractive absorption properties in the UV–Vis region. This capability has been widely described in the literature where cyano­acrylates were employed as precursors for the synthesis of dye-sensitized photovoltaic materials (Chen *et al.*, 2013 ▸; Zietz *et al.*, 2014 ▸; Lee *et al.*, 2009 ▸) and sensors (Zhang *et al.*, 2010 ▸). Considering that the absorption properties are related to the mol­ecular structure of cyano­acrylate compounds (Ma *et al.*, 2014 ▸), it is therefore very useful to know their crystal structures in detail in order to have a better understanding of the link between the structures and properties of these derivatives. In this contribution, we present the crystal structure of a thio­phene-based cyano­acrylate derivative with promising applications in the synthesis of ligands for metal sensing.
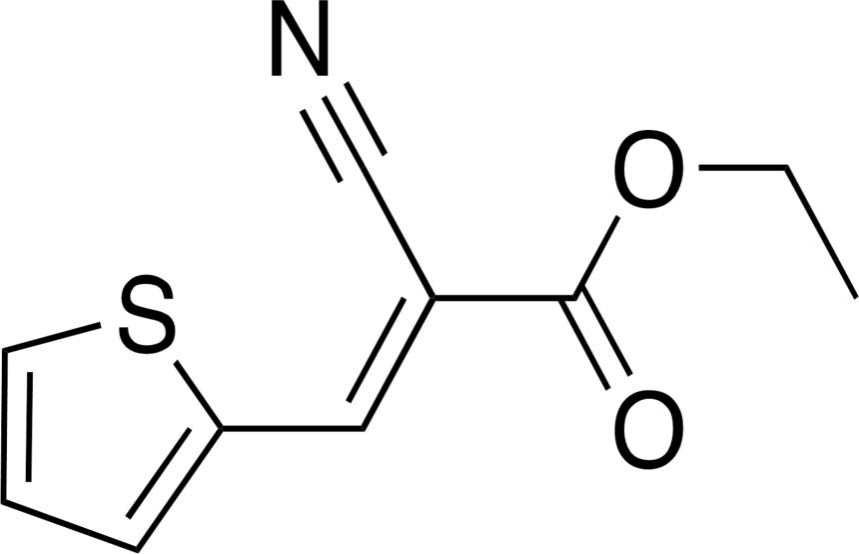


## Structural commentary   

**Figure 1:**
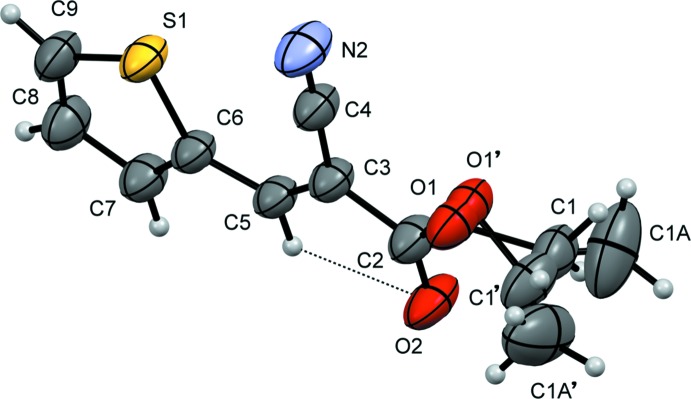
The mol­ecular structure of the title compound, showing anisotropic displacement ellipsoids drawn at the 50% probability level. The intra­molecular C—H⋯O hydrogen bond is shown as a dashed line (see Table 1 ▸) and the discrete disorder in the ethyl moiety is also observed.

Fig. 1 ▸ shows the mol­ecule of the title compound. The near planarity of the mol­ecule (r.m.s. deviation of 0.006 Å) means that nearly all atoms lie in the same plane perpendicular to [010] except for the ethyl ester fragment (O2/C2/O1/C1/C1*A*), which presents a discrete disorder due to the existence of two conformations of the ethyl moiety that overlay in the same crystallographic site. This disorder was modelled using two sites for the O1, C1 and C1*A* atoms with occupancy values of 0.5. The split fragment is observed as a reflection of two ethyl moieties in the two opposite sides of the mirror plane that contains the mol­ecule. These atoms lie, respectively, 0.21 (2), 0.340 (7) and −1.010 (10) Å out of this plane. The planarity allows the formation of a weak intra­molecular C5—H5⋯O2 close contact (Fig. 1 ▸ and Table 1 ▸), which generates an *S*(6) motif. This mol­ecule is similar to (*E*)-ethyl-2-cyano-3-(furan-2-yl)acrylate (Kalkhambkar *et al.*, 2012 ▸), differing in the five-membered ring, which is a furanyl in this compound, and presenting a distorted planarity compared with the title compound [dihedral angles of 177.5–179.0° in the two molecules of the asymmetric unit compared with the value of 180.0° in the C6-C5-C3-C2 fragment of the title compound]. Also, no mol­ecular disorder was reported in the furanyl mol­ecule.

## Supra­molecular features   

**Figure 2:**
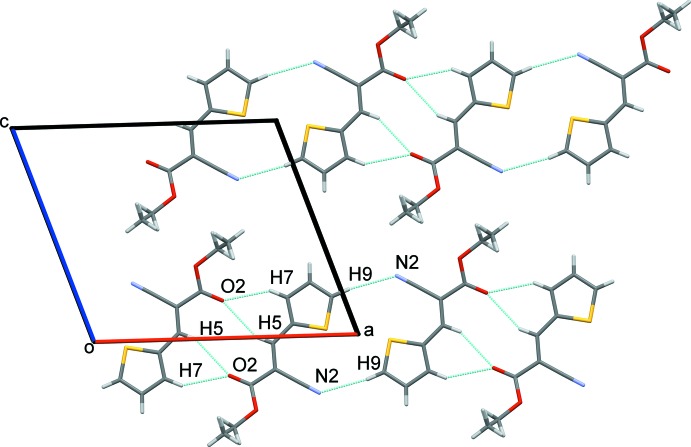
The crystal structure of the title compound, showing the C—H⋯(O, N) hydrogen-bonding inter­actions (dotted lines) along the [100] direction.

In the crystal, the packing is directed by C5—H5⋯O2^i^ and C7—H7⋯O2^i^ [symmetry code: (i) −*x* + 1, −*y* + 1, −*z*] (see Table 1 ▸ and Fig. 2 ▸) inter­actions, which connect pairs of inversion-related mol­ecules, forming slabs of infinite chains running along [100] with 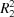
(10) and 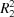
(14) motifs, respectively (see Fig. 2 ▸). These slabs are further linked by weak C9—H9⋯N2^ii^ [symmetry code: (ii) −*x*, −*y* + 1, −*z*] inter­actions along the *a*-axis direction (Table 1 ▸). Neighboring chains inter­act along [001] direction by van der Waals forces, forming (010) sheets. In the [010] direction, only weak dipolar inter­actions or van der Waals forces act between neighboring sheets to consolidate the three-dimensional array of the crystal structure. Despite the mol­ecular similarity with (*E*)-ethyl-2-cyano-3-(furan-2-yl)acrylate (Kalkhambkar *et al.*, 2012 ▸), the inversion-related molecules in Kalkhambkar’s structure, joined by similar intermolecular hydrogen bonds, are further connected by different sorts of C—H⋯O and C—H⋯N weaker interactions involving the furanyl ring.

## Database survey   

A search of the Cambridge Structural Database (CSD Version 5.37 with two updates, Groom *et al.*, 2016 ▸) for the complete mol­ecule given the option for any substituent in the five-membered ring and/or allowing a saturated chain longer than the ethyl fragment gave three hits, all of them forming parts of mol­ecules bigger than the title compound, giving different supra­molecular inter­actions due not only to the loss of planarity, as in the case of the ethyl-3-(3-chloro-4-cyano-5-{[4-(di­methyl­amino)­phen­yl]diazen­yl}-2-thien­yl)-2-cyano­acrylate (Xu *et al*., 2016 ▸), but also due to an increase in the saturated chains as in the case of octyl-2-cyano-3-(4,6-di­bromo-7,7-dimethyl-7*H*-thieno[3′,4′:4,5]silolo[2,3-*b*]thio­phen-2-yl)acryl­ate (Liu *et al*., 2016 ▸) and ethyl-2-cyano-3-(3,3′′′-dihexyl-2,2′:5′,2′′:5′′,2′′′-quaterthio­phen-5-yl)acrylate (Miyazaki *et al.*, 2011 ▸). A search considering any heteroatom in the place of S1 gave six hits. Among them, the more similar compounds correspond to ethyl-(2*E*)-2-cyano-3-(1-methyl-1*H*-pyrrol-2-yl)prop-2-enoate (Asiri *et al.*, 2011 ▸), (*E*)-ethyl-2-cyano-3-(1*H*-pyrrol-2-yl)acrylate (Yuvaraj *et al.*, 2011 ▸) and (*E*)-ethyl-2-cyano-3-(furan-2-yl)acrylate (Kalkhambkar *et al.*, 2012 ▸), the last one being the most similar compound since its mol­ecular conformation is also planar, with the ethyl fragment out of the plane and a furanyl forming the five-membered ring.

## Synthesis and crystallization   

**Figure 3:**
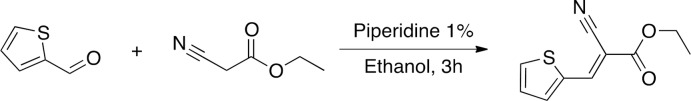
Schematic representation of the synthetic pathway of ethyl (*E*)-2-cyano-3-(thio­phen-2-yl)acrylate.

All reagents and solvents were purchased from commercial sources and used as received. In a two-necked round-bottom flask equipped with a condenser, thio­phene-2-carboxaldehyde (740 mg, 6.6 mmol), cyano­acetic acid ethyl ester (753 mg, 6.6 mmol) and piperidine (6,8 µL, 1% mol) were stirred in ethanol for three h. A yellowish brown solid was obtained and recrystallized from ethanol solution (see Fig. 3 ▸). The product was filtered out and then dried under vacuum. The yellowish brown solid was dissolved in methanol and yellow crystals were grown through slow evaporation of the solvent at room temperature with 80% yield. Melting point: 366–367 K, reported: 365–367 K (Jia *et al.* 2015 ▸). ^1^H NMR: (DMSO-*d* ^6^, 400 MHz, *d*, ppm): 1,41 (*t*, 2H), 4,38 (*q*, 3H), 7.25 (*dd*, 1H), 7,81 (*d*, 1H), 7,85 (*d*, 1H), 8.36 (*s*, 1H). ^13^C NMR (DMSO-*d^6^*, 100 MHz, *d*, ppm): 14.19, 62.54, 99.3, 115.6, 128.6, 135.1, 136.1, 137.1, 146.6, 162.8.

## Refinement   

Crystal data, data collection and structure refinement details are summarized in Table 2 ▸. H atoms were placed in calculated positions (C—H: 0.93–0.97 Å) and included as riding contributions with isotropic displacement parameters set at 1.2–1.5 times the *U*_eq_ value of the parent atom.

## Supplementary Material

Crystal structure: contains datablock(s) I. DOI: 10.1107/S2056989017010799/ff2150sup1.cif

Structure factors: contains datablock(s) I. DOI: 10.1107/S2056989017010799/ff2150Isup2.hkl

Supporting information file. DOI: 10.1107/S2056989017010799/ff2150Isup3.cml

CCDC reference: 1563845

Additional supporting information:  crystallographic information; 3D view; checkCIF report

## Figures and Tables

**Table 1:** Hydrogen-bond geometry (Å, °)

*D*—H⋯*A*	*D*—H	H⋯*A*	*D*⋯*A*	*D*—H⋯*A*
C5—H5⋯O2	0.93	2.42	2.799 (3)	104
C7—H7⋯O2^i^	0.93	2.55	3.363 (3)	147
C5—H5⋯O2^i^	0.93	2.57	3.425 (3)	153
C9—H9⋯N2^ii^	0.93	2.60	3.520 (4)	172

Symmetry codes: (i) 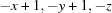; (ii) 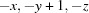.

**Table 2:** Experimental details

Crystal data	
Chemical formula	C_10_H_9_NO_2_S
*M*_r_	207.24
Crystal system, space group	Monoclinic, *C*2/*m*
Temperature (K)	298
*a*, *b*, *c* (Å)	13.637 (2), 6.8965 (16), 11.817 (3)
β (°)	109.28 (2)
*V* (Å^3^)	1049.0 (4)
*Z*	4
Radiation type	Mo *K*α
μ (mm^−1^)	0.28
Crystal size (mm)	0.19 × 0.12 × 0.07

Data collection	
Diffractometer	Agilent SuperNova, Dual, Cu at zero, Atlas
Absorption correction	Multi-scan (*CrysAlis PRO*; Agilent, 2014 ▸)
*T*_min_, *T*_max_	0.760, 1.000
No. of measured, independent and observed [*I* > 2σ(*I*)] reflections	9896, 1171, 1049
*R*_int_	0.068
(sin θ/λ)_max_ (Å^−1^)	0.625

Refinement	
*R*[*F* ^2^ > 2σ(*F* ^2^)], *wR*(*F* ^2^), *S*	0.047, 0.126, 1.14
No. of reflections	1171
No. of parameters	96
H-atom treatment	H-atom parameters constrained
Δρ_max_, Δρ_min_ (e Å^−3^)	0.35, −0.24

Computer programs: *CrysAlis PRO* (Agilent, 2014 ▸), *SUPERFLIP* (Palatinus & Chapuis, 2007 ▸), *SHELXL2014* (Sheldrick, 2015 ▸) and *Mercury* (Macrae *et al.*, 2008 ▸).

## supplementary crystallographic information

### Crystal data

**Table:**

C_10_H_9_NO_2_S	*F*(000) = 432
*M**_r_* = 207.24	*D*_x_ = 1.312 Mg m^−^^3^
Monoclinic, *C*2/*m*	Mo *K*α radiation, λ = 0.71073 Å
*a* = 13.637 (2) Å	Cell parameters from 2818 reflections
*b* = 6.8965 (16) Å	θ = 4.5–26.3°
*c* = 11.817 (3) Å	µ = 0.28 mm^−^^1^
β = 109.28 (2)°	*T* = 298 K
*V* = 1049.0 (4) Å^3^	Parallelepiped, yellow
*Z* = 4	0.19 × 0.12 × 0.07 mm

### Data collection

**Table:**

Agilent SuperNova, Dual, Cu at zero, Atlas diffractometer	1171 independent reflections
Radiation source: SuperNova (Mo) X-ray Source	1049 reflections with *I* > 2σ(*I*)
Detector resolution: 5.3072 pixels mm^-1^	*R*_int_ = 0.068
ω scans	θ_max_ = 26.4°, θ_min_ = 3.1°
Absorption correction: multi-scan (CrysAlis PRO; Agilent, 2014)	*h* = −16→16
*T*_min_ = 0.760, *T*_max_ = 1.000	*k* = −8→8
9896 measured reflections	*l* = −14→14

### Refinement

**Table:**

Refinement on *F*^2^	Secondary atom site location: difference Fourier map
Least-squares matrix: full	Hydrogen site location: inferred from neighbouring sites
*R*[*F*^2^ > 2σ(*F*^2^)] = 0.047	H-atom parameters constrained
*wR*(*F*^2^) = 0.126	*w* = 1/[σ^2^(*F*_o_^2^) + (0.053*P*)^2^ + 0.7374*P*] where *P* = (*F*_o_^2^ + 2*F*_c_^2^)/3
*S* = 1.14	(Δ/σ)_max_ < 0.001
1171 reflections	Δρ_max_ = 0.35 e Å^−^^3^
96 parameters	Δρ_min_ = −0.24 e Å^−^^3^
0 restraints	Extinction correction: SHELXL2016 (Sheldrick, 2016), Fc^*^=kFc[1+0.001xFc^2^λ^3^/sin(2θ)]^-1/4^
Primary atom site location: iterative	Extinction coefficient: 0.007 (2)

### Special details

**Table:**

Geometry. All esds (except the esd in the dihedral angle between two l.s. planes) are estimated using the full covariance matrix. The cell esds are taken into account individually in the estimation of esds in distances, angles and torsion angles; correlations between esds in cell parameters are only used when they are defined by crystal symmetry. An approximate (isotropic) treatment of cell esds is used for estimating esds involving l.s. planes.

### Fractional atomic coordinates and isotropic or equivalent isotropic displacement parameters (Å^2^)

**Table:**

	*x*	*y*	*z*	*U*_iso_*/*U*_eq_	Occ. (<1)
S1	0.11266 (5)	0.500000	−0.02961 (7)	0.0560 (3)	
N2	0.2293 (2)	0.500000	0.2653 (2)	0.0733 (9)	
O2	0.53999 (15)	0.500000	0.1743 (2)	0.0732 (7)	
C2	0.4730 (2)	0.500000	0.2195 (3)	0.0568 (7)	
C3	0.36045 (19)	0.500000	0.1505 (2)	0.0480 (6)	
C4	0.2879 (2)	0.500000	0.2153 (3)	0.0531 (7)	
C7	0.2120 (2)	0.500000	−0.1795 (3)	0.0555 (7)	
H7	0.264802	0.500000	−0.213002	0.067*	
C6	0.22929 (19)	0.500000	−0.0583 (2)	0.0470 (6)	
C5	0.33082 (19)	0.500000	0.0298 (2)	0.0468 (6)	
H5	0.385193	0.500000	−0.001307	0.056*	
C8	0.1056 (2)	0.500000	−0.2475 (3)	0.0630 (8)	
H8	0.080614	0.500000	−0.330873	0.076*	
C9	0.0436 (2)	0.500000	−0.1786 (3)	0.0616 (8)	
H9	−0.028547	0.500000	−0.209250	0.074*	
O1	0.48964 (18)	0.531 (3)	0.3371 (2)	0.064 (3)	0.5
C1	0.5976 (3)	0.5493 (10)	0.4143 (4)	0.073 (3)	0.5
H1A	0.600898	0.615277	0.487917	0.088*	0.5
H1B	0.636145	0.625544	0.374142	0.088*	0.5
C1A	0.6446 (5)	0.3535 (15)	0.4422 (6)	0.127 (3)	0.5
H1AA	0.641144	0.288738	0.369077	0.191*	0.5
H1AB	0.607205	0.279578	0.483442	0.191*	0.5
H1AC	0.715902	0.365498	0.492230	0.191*	0.5

### Atomic displacement parameters (Å^2^)

**Table:**

	*U*^11^	*U*^22^	*U*^33^	*U*^12^	*U*^13^	*U*^23^
S1	0.0331 (4)	0.0805 (6)	0.0559 (5)	0.000	0.0168 (3)	0.000
N2	0.0468 (14)	0.122 (3)	0.0570 (16)	0.000	0.0246 (12)	0.000
O2	0.0353 (10)	0.130 (2)	0.0567 (13)	0.000	0.0184 (9)	0.000
C2	0.0379 (14)	0.082 (2)	0.0506 (16)	0.000	0.0144 (12)	0.000
C3	0.0353 (13)	0.0627 (16)	0.0477 (15)	0.000	0.0161 (11)	0.000
C4	0.0373 (13)	0.0740 (19)	0.0472 (15)	0.000	0.0127 (12)	0.000
C7	0.0428 (14)	0.0724 (19)	0.0527 (16)	0.000	0.0179 (12)	0.000
C6	0.0334 (12)	0.0580 (15)	0.0512 (15)	0.000	0.0161 (11)	0.000
C5	0.0332 (12)	0.0559 (15)	0.0530 (15)	0.000	0.0167 (11)	0.000
C8	0.0491 (16)	0.088 (2)	0.0462 (16)	0.000	0.0074 (12)	0.000
C9	0.0368 (14)	0.079 (2)	0.0622 (18)	0.000	0.0069 (13)	0.000
O1	0.0410 (11)	0.103 (10)	0.0458 (12)	−0.003 (2)	0.0122 (9)	−0.008 (2)
C1	0.046 (2)	0.112 (9)	0.055 (2)	−0.005 (2)	0.0070 (17)	−0.019 (3)
C1A	0.093 (5)	0.188 (9)	0.077 (4)	0.053 (5)	−0.004 (3)	−0.019 (5)

### Geometric parameters (Å, º)

**Table:**

S1—C9	1.700 (3)	C5—H5	0.9300
S1—C6	1.732 (3)	C8—C9	1.354 (5)
N2—C4	1.139 (4)	C8—H8	0.9300
O2—C2	1.200 (3)	C9—H9	0.9300
C2—O1	1.350 (5)	O1—C1	1.459 (5)
C2—C3	1.482 (4)	C1—C1A	1.485 (11)
C3—C5	1.347 (4)	C1—H1A	0.9700
C3—C4	1.437 (4)	C1—H1B	0.9700
C7—C6	1.372 (4)	C1A—H1AA	0.9600
C7—C8	1.407 (4)	C1A—H1AB	0.9600
C7—H7	0.9300	C1A—H1AC	0.9600
C6—C5	1.431 (4)		
			
C9—S1—C6	91.57 (14)	C9—C8—H8	123.6
O2—C2—O1	124.3 (3)	C7—C8—H8	123.6
O2—C2—C3	123.9 (3)	C8—C9—S1	112.4 (2)
O1—C2—C3	111.0 (2)	C8—C9—H9	123.8
C5—C3—C4	123.0 (2)	S1—C9—H9	123.8
C5—C3—C2	118.5 (2)	C2—O1—C1	116.7 (3)
C4—C3—C2	118.5 (2)	O1—C1—C1A	109.5 (8)
N2—C4—C3	179.1 (3)	O1—C1—H1A	109.8
C6—C7—C8	112.7 (3)	C1A—C1—H1A	109.8
C6—C7—H7	123.6	O1—C1—H1B	109.8
C8—C7—H7	123.6	C1A—C1—H1B	109.8
C7—C6—C5	123.4 (2)	H1A—C1—H1B	108.2
C7—C6—S1	110.6 (2)	C1—C1A—H1AA	109.5
C5—C6—S1	126.0 (2)	C1—C1A—H1AB	109.5
C3—C5—C6	130.5 (3)	H1AA—C1A—H1AB	109.5
C3—C5—H5	114.7	C1—C1A—H1AC	109.5
C6—C5—H5	114.7	H1AA—C1A—H1AC	109.5
C9—C8—C7	112.8 (3)	H1AB—C1A—H1AC	109.5
			
O2—C2—C3—C5	0.000 (1)	C2—C3—C5—C6	180.000 (1)
O1—C2—C3—C5	170.1 (8)	C7—C6—C5—C3	180.000 (1)
O2—C2—C3—C4	180.000 (1)	S1—C6—C5—C3	0.000 (1)
O1—C2—C3—C4	−9.9 (8)	C6—C7—C8—C9	0.000 (1)
C8—C7—C6—C5	180.000 (1)	C7—C8—C9—S1	0.000 (1)
C8—C7—C6—S1	0.000 (1)	C6—S1—C9—C8	0.000 (1)
C9—S1—C6—C7	0.000 (1)	O2—C2—O1—C1	−5.6 (17)
C9—S1—C6—C5	180.0	C3—C2—O1—C1	−175.6 (9)
C4—C3—C5—C6	0.000 (1)	C2—O1—C1—C1A	−79.5 (13)

### Hydrogen-bond geometry (Å, º)

**Table:**

*D*—H···*A*	*D*—H	H···*A*	*D*···*A*	*D*—H···*A*
C5—H5···O2	0.93	2.42	2.799 (3)	104
C7—H7···O2^i^	0.93	2.55	3.363 (3)	147
C5—H5···O2^i^	0.93	2.57	3.425 (3)	153
C9—H9···N2^ii^	0.93	2.60	3.520 (4)	172

Symmetry codes: (i) −*x*+1, −*y*+1, −*z*; (ii) −*x*, −*y*+1, −*z*.
